# Ultrastructural analyses of the novel chimeric hemostatic agent generated via nanotechnology, ABS nanohemostat, at the renal tissue level

**DOI:** 10.1186/s40064-016-3625-z

**Published:** 2016-11-08

**Authors:** Emre Huri, Engin Dogantekin, Murvet Hayran, Umit Yavuz Malkan, Mine Ergun, Aysegul Firat, Yavuz Beyazit, Huseyin Ustun, Murat Kekilli, Mumtaz Dadali, Muzeyyen Astarci, Ibrahim C. Haznedaroglu

**Affiliations:** 1 Department of Urology, Hacettepe University Medical School, Ankara, Turkey; 2 Department of Anatomy, Hacettepe University Medical School, Ankara, Turkey; 3 Department of Hematology, Hacettepe University Medical School, Ankara, Turkey; 4 Department of Gastroenterology, Turkiye Yuksek Ihtisas Training and Research Hospital, Ankara, Turkey; 5 Department of Pathology, Ankara Training and Research Hospital, Ankara, Turkey

**Keywords:** Ankaferd nanohemostat, Ultrastructural analysis, Safety, Renal tissue

## Abstract

Ankaferd Blood Stopper (ABS), a hemostatic agent of plant origin, has been registered for the prevention of clinical hemorrhages. Currently there is no data regarding the ultrastructural analysis of ABS at the tissue level. The aim of this study is to assess renal tissue effects via scanning electron microscopy (SEM) analyses for the ABS and ABS nanohemostat (formed by the combination of self-assembling peptide amphiphile molecules and ABS). SEM experiments were performed with FEI Nova NanoSEM 230, using the ETD detector at low vacuum mode with 30 keV beam energy. SEM analyses revealed that significant erythroid aggregation are present inside the capillary bed of the renal tissue. However, neither the signs of necrosis nor any other sign of tissue damage are evident in the surrounding renal tissue supplied by the microcapillary vasculature. Our study is important for several reasons. Firstly, in our study we used ABS nanohemostat which was recently developed. This study adds valuable information to the literature regarding ABS nanohemostat. Secondly, this study is the first ultrastructural analysis of ABS that was performed at the tissue level. Thirdly, we disclosed that ABS nanohemostat could induce vital erythroid aggregation at the renal tissue level as detected by SEM. Lastly, we detected that ABS nanohemostat causes no harm to the tissues including necrosis and any other detrimental effects.

## Background

Ankaferd Blood Stopper (ABS), is a hemostatic agent of plant origin, that has been registered for the prevention of clinical hemorrhages when the other techniques are ineffective (Beyazit et al. 2010, 2011; Haznedaroglu et al. 2012; Goker et al. 2007). The ABS effects occurred via the protein agglutination and polymerization could modify vital erythroid aggregation inside vascular endothelium (Haznedaroglu et al. 2012). The clinical efficacy of ABS in numerous systems had been demonstrated in the literature. For instance; ABS is very effective in the controlling of hemorrhages within the gingival tissue (Amer et al. 2014), lung tissue (Uzun et al. 2014), tooth extraction (Kazancıoğlu et al. 2013; Tek et al. 2014; Beyazit et al. 2011), perineal tissue (Eyi et al. 2013), oral mucosa tissue (Leblebisatan et al. 2012), gastrointestinal tissue (Karaman et al. 2012), thyroid tissue (Guler et al. 2011), nasopharyngeal tissue (Iynen et al. 2011; Yasar and Ozkul 2011), nasal tissue (Teker et al. 2010), peritonsillary tissue (Teker et al. 2009). Also, it has been reported that partial nephrectomy operation in human was performed without any complication with ABS (Huri et al. 2010). ABS is shown to be effective and safely used in the emergent beating heart coronary artery bypass grafting (Atalay et al. 2015). Moreover, ABS could control the hemorrhages in the cases who were using antiagregant treatment and undergone emergency bypass operation (Akpinar et al. 2015). Besides those valuable data for the efficacy of ABS, there is no available data regarding the ultrastructural analysis of ABS at tissue level.

ABS nanohemostat is a chimeric hemostatic agent. The medicine was developed by combining a self-assembling peptide amphiphile (PA) molecule with the traditional ABS (Huri et al. 2013). ABS nanohemostat is useful in particular cases such as partial nephrectomy because traditional ABS could not be so effective due to insufficient contact surface between the ABS hemostatic liquid agent and the bleeding area (Huri et al. 2013). Therefore the main function of PA is to carry active ABS agent onto the tissues in order to facilitate hemostatic action in a given tissue.

The aim of this study is to assess renal tissue effects via Scanning Electron Microscopy (SEM) analyses for the ABS nanohemostat formed by the combination of self-assembling peptide amphiphile (PA) molecules and ABS. Elucidation of the safety of ABS nanohemostat is extremely important at the tissue level since Ankaferd has already been utilized in a vast variety of human clinical trials such as heart surgery, tonsillectomy, thyroidectomy, episiotomy and many others.

## Methods

### Animals and materials

The specimens were acquired from previously performed study by our group (Huri et al. 2013). All animal experimentations described in this paper were carried out in accordance with national guidelines for the use and care of laboratory animals and were approved by the local animal review and “Ankara Hospital Animal Study Ethics Committee, Ankara/Turkey”. All procedures were in full compliance with Turkish Law 6343/2, Veterinary Medicine Deontology Regulation 6.7.26, and with the Helsinki Declaration of World Medical Association recommendations on animal studies. The animals were obtained from the center of medical experimental research of Ankara Training and Research Hospital. The rats were housed in stainless steel cages in an animal room maintained at a temperature of 22–24 °C with 12-h light/dark periods. All were fed with the same amount of laboratory pellet diet and with water supplied ad libitum for a minimum of 5 days before partial nephrectomy (PN). In the previously published study by our group, 9-Fluorenylmethoxycarbonyl (Fmoc), ter. Butoxycarbonyl (Boc) protected amino acids, Rink Amide MBHA resin and 2-(1*H*-Benzotriazol-1-yl)-1,1,3,3-tetramethyluronium hexafluorophosphate (HBTU) were purchased from NovaBiochem or ABCR. The other chemicals were purchased from Fisher, Merck, Alfa Aesar or Aldrich and used as provided. A total of 24 Wistar rats weighing 200–300 g were divided into 4 groups of 6 each and underwent PN. One surgeon with an assistant performed all the surgical procedures. All operations were performed under general anaesthesia with injection of 50 mg/kg intramuscular ketamine hydrochloride. After sterile preparation and draping, a midline incision was made on the abdomen. For each rat, renal artery and vein were exposed by hilar vascular dissection. Subsequently renal artery and vein were clamped with Rommel vascular clamp. The lower third of the left kidney was resected in guillotine fashion with a single stroke of an amputating knife. Four different hemostatic techniques were applied to the chronological groups.

- Group 1 (G1): Left PN with hilar vascular control including intracorporeal suturing of the renal parenchyma and collecting duct.
- Group 2 (G2): Conventional PN with only 0.5 ml traditional Ankaferd hemostat (ABS) application without suturing.
- Group 3 (G3): Conventional PN with ABS (0.25 ml) + peptide (0.25 ml) gel (ABS Nanohemostat) mixture application with no suturing.
- Group 4 (G4): Conventional PN with only 0.5 ml peptide solution application.

Two objective parameters were recorded during the surgical procedure: warm ischemia time (WIT) and amount of bleeding (AOB). The unit of WIT was the “second” while the AOB was measured with the bleeding area (cm^2^) onto the sponges. After the procedures, the sponges were used to collect all visible clots and blood to be measured for the bleeding surface detection. The abdominal incision was afterwards closed with surgical sutures. All the rats were allowed to feed and drink water for the following 4 weeks. After then, each rat was sacrificed and total nephrectomy was performed for histopathological examination.

### The hemostatic methods during PN

Each hemostatic method was used during the period of warm ischemia (WI). WI started with clamping the renal artery and vein, and finished with taking the clamp out. In G1, traditional hemostasis method was used as compression onto the renal excised area and suturing the renal vessels and collecting duct with absorbable sutures. In G2, 2 ml of ABS was dropped to the amputated renal margin steadily until bleeding stopped. In G3, ABS (2 ml) + Nanopeptide gel mixture (ABS nanohemostat) was applied onto the injured area. And in G4, only nanopeptide gel was used to control bleeding. Other hemostatic methods including sponges, Surgicel, electrocautery and any other sources were not used to control bleeding in the present study.

### Scanning electron microscopy (SEM) imaging of rat kidney tissues

The portions of the kidneys from the rats in the studied groups were cut into small blocks, immersed and rinsed to be examined in the SEM analyses. The SEM experiments were performed with FEI Nova NanoSEM 230, using the ETD detector at low vacuum mode with 30 keV beam energy.

## Results

Warm ischemia time for each group was 232.8 ± 56.3, 65.6 ± 11.4, 75.5 ± 17.2, 58.1 ± 17.6 s in Group 1,2,3 and 4, respectively (p:0.003). The amount of bleeding for each group was 7.3 ± 3.3, 5.7 ± 2.3, 5.2 ± 3.2, 16.4 ± 7.7 cm^2^ in Group 1, 2, 3 and 4, respectively (p: 0.035).

**Fig. 1:**
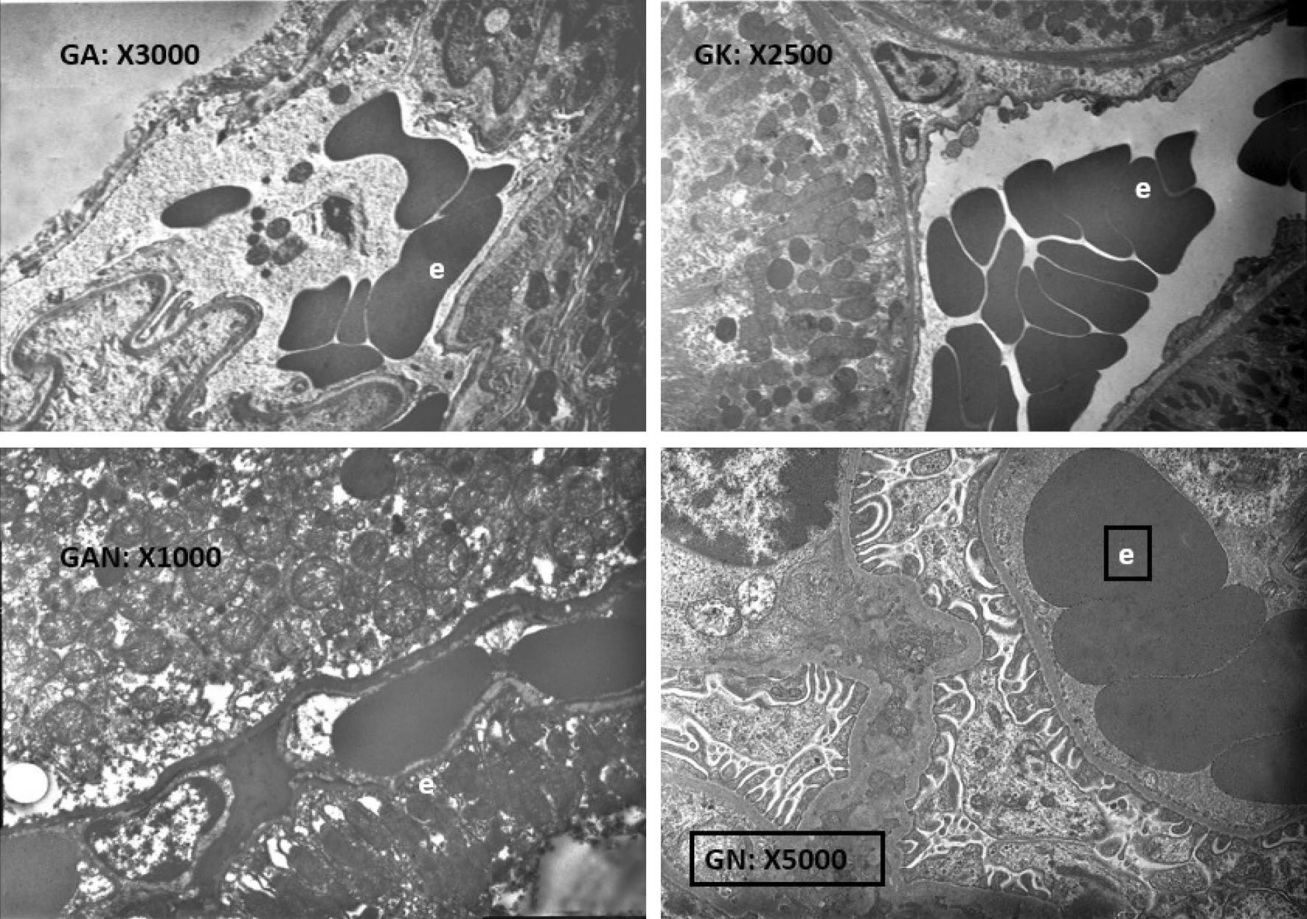
Red blood cell aggregation are present inside the capillary bed of the renal tissue upon the application of Ankaferd hemostat. The appearance of the nucleus, cytoplasm of the vascular endothelial cells and their organelles are completely normal (*e* erythrocyte)

The SEM analyses of the G2 (Conventional PN with only 0.5 ml traditional Ankaferd hemostat (ABS) application without suturing) and G3 (Conventional PN with ABS (0.25 ml) + peptide (0.25 ml) gel (ABS Nanohemostat) mixture application with no suturing) revealed that significant erythroid aggregation are present inside the capillary bed of the renal tissue upon the application of both ABS and ABS nanohemostat. However, neither the signs of necrosis nor any other sign of tissue damage are evident in the surrounding renal tissue supplied by the microcapillary vasculature in both ABS and ABS nanohemostat. Furthermore, the appearance of the nucleus, cytoplasm of the vascular endothelial cells and their organelles are completely normal with both agents (Fig. 1).

**Fig. 2:**
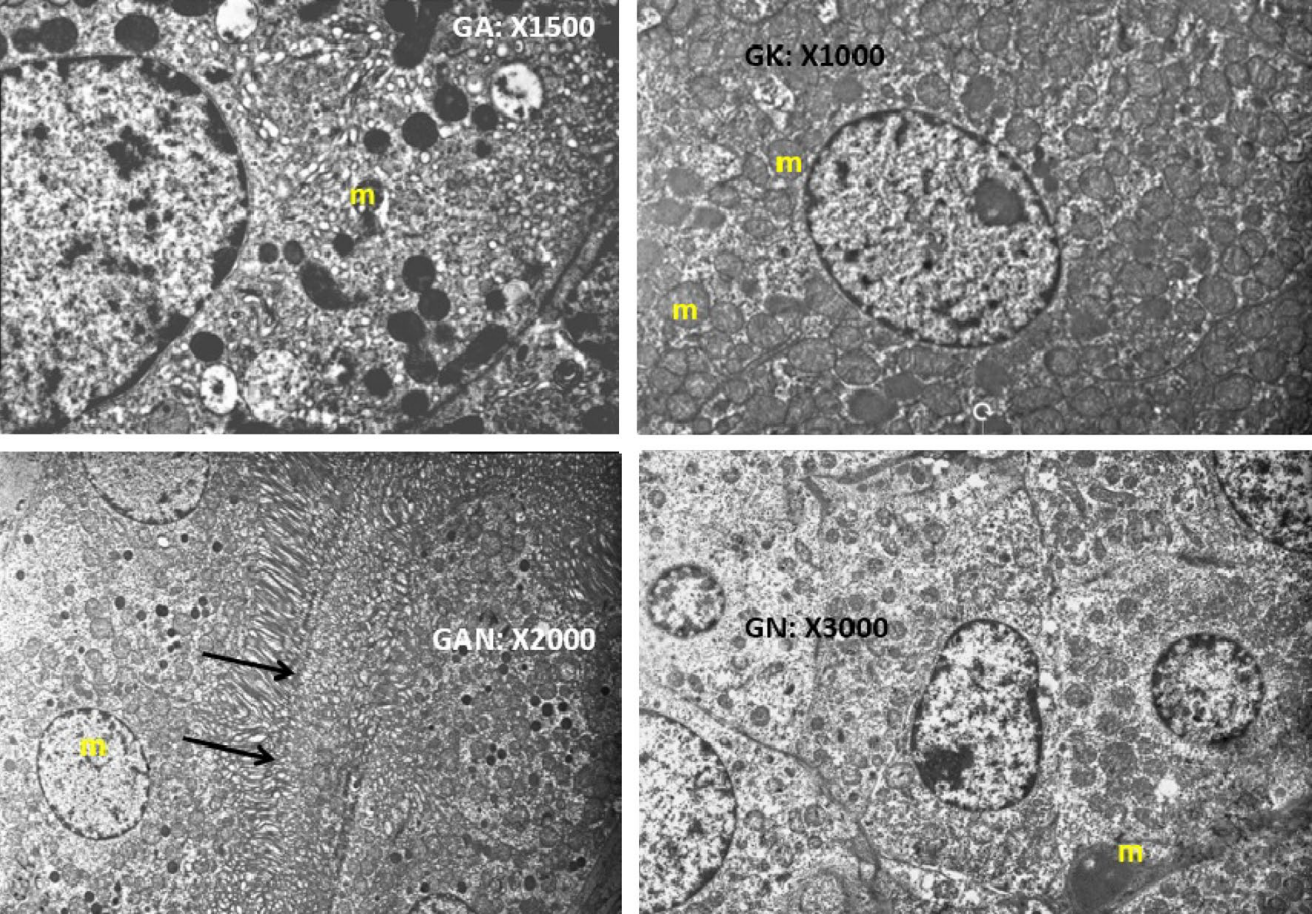
The nucleus of cells, the cell membrane and intracellular organelles have normal view which is observed in each level at the Ankaferd hemostat applied renal tissue. Proximal tubule cells are observed which constitutes a large part of the cortical parenchyma. There are a large number of normal-looking mitochondria in the cytoplasm of cells which is lining the proximal tubules. Many vesicles are observed in the cytoplasm which is located in the apical portion of the cells. A large number of normal-looking microvilli creates brush border and is viewed on the apical cell membrane of the proximal tubule (*m* mitochondria, *black arrows* microvilli)

In the SEM analyses of the both research groups of G2 and G3, proximal tubule cells are observed which constitutes a large part of the cortical parenchyma. The nucleus of cells, the cell membrane and intracellular organelles have normal view which is observed in each level (Fig. 2). There are a large number of normal-looking mitochondria in the cytoplasm of cells which is lining the proximal tubules. Many vesicles are observed in the cytoplasm which is located in the apical portion of the cells. A large number of normal-looking microvilli creates brush border and is viewed on the apical cell membrane of the proximal tubule. Topical ABS application had no negative effects to the renal parenchyma at the level of renal cortex.

**Fig. 3:**
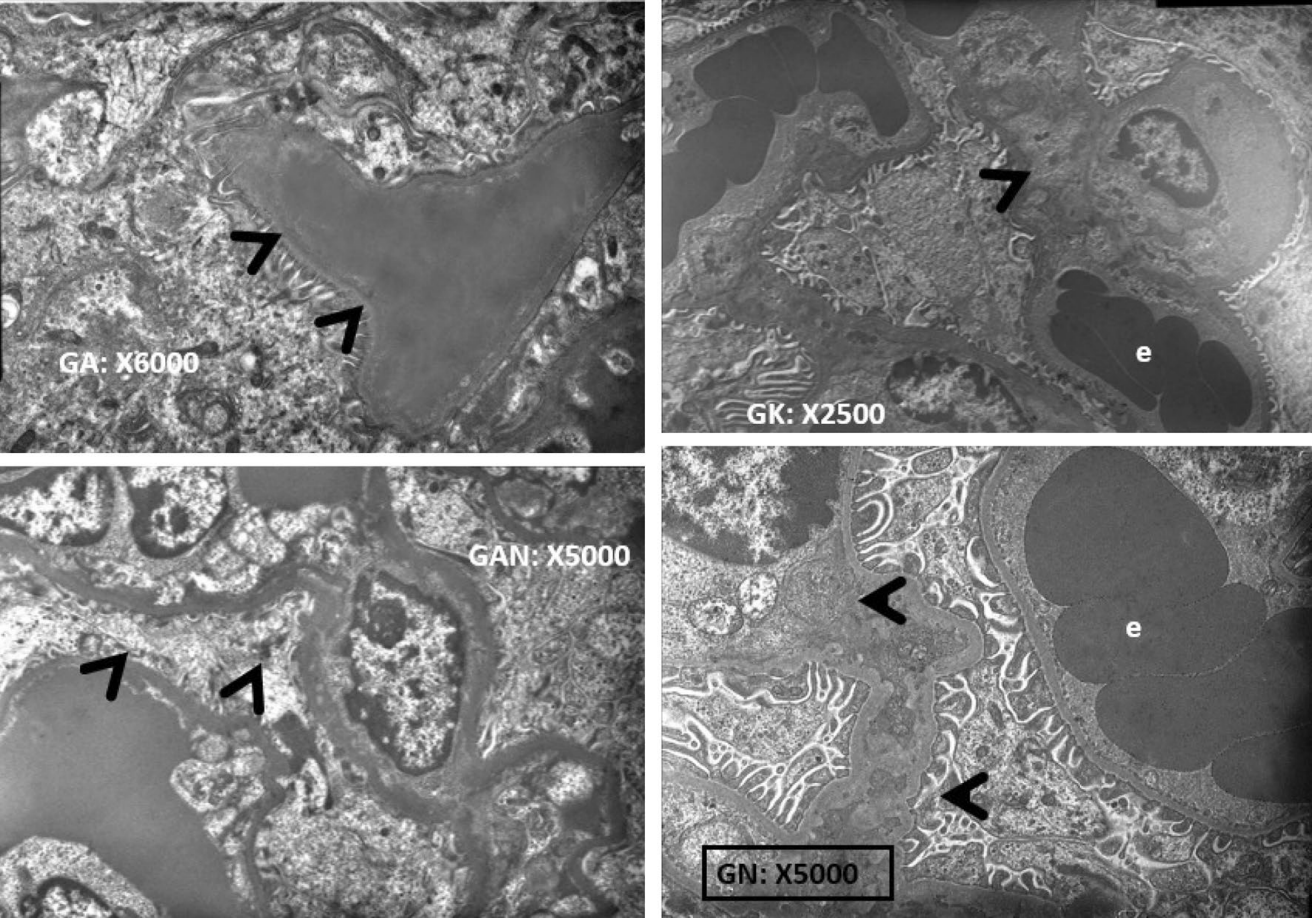
In the glomerular capillary endothelium, fenestrated endothelial cells, basement membrane and tripartite structure created by the podocytes viewed as completely normal (*e* erythrocyte, *black arrows* podocytes)

In the SEM analyses of the both research groups of G2 and G3, in the glomerular capillary endothelium, fenestrated endothelial cells, basement membrane and tripartite structure created by the podocytes viewed completely normal (Fig. 3). The mesangial cells are also localized in mesangium was normal. The damage was not present on the glomerules, mesangial cells and critique renal tissues like as endothelial structures. Neither ABS nor ABS nanohemostat caused any damage at the level of cell and intracellular organelles in the kidneys.

**Fig. 4:**
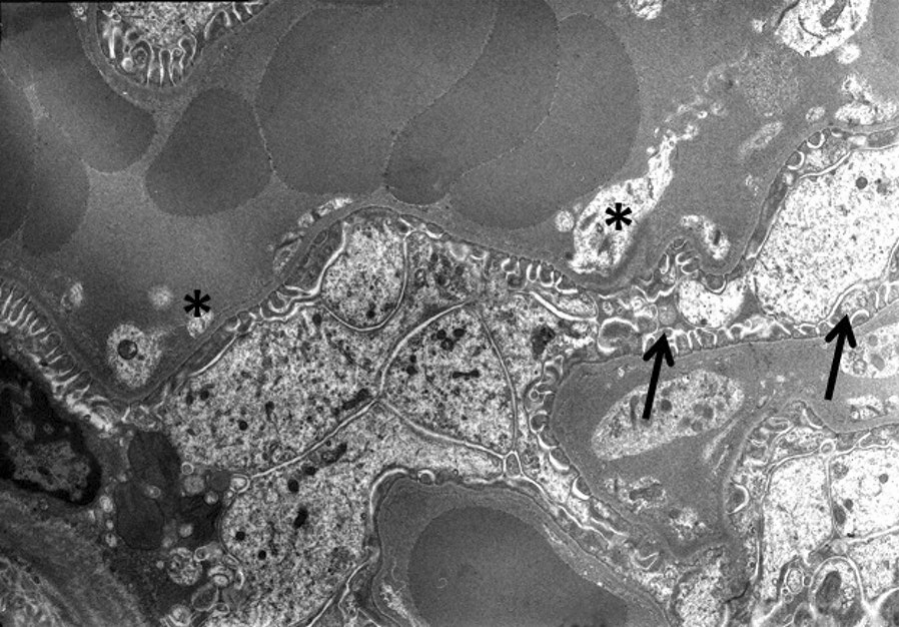
Glomerulus are generally normal, fenestrates are spacious in some place of basal lamina on the side of the endothel and edema present. Pedicels are seen normal (*black arrows*; fenestrates, * pedicels)

**Fig. 5:**
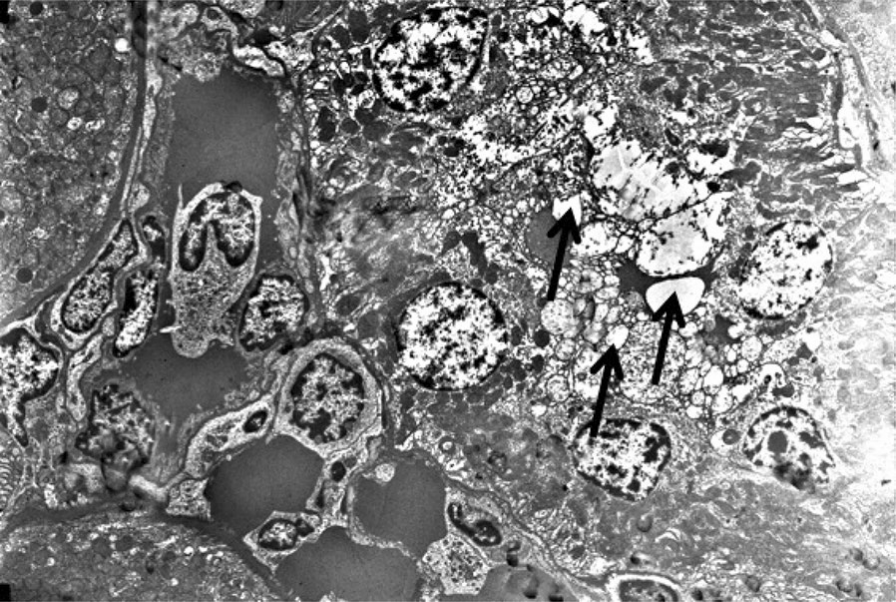
Though proximal tubules are seen normal in most of the field, intracellular edema is seen in some places (*black arrows* edema)

The SEM analyses of the G1 (Left PN with hilar vascular control including intracorporeal suturing of the renal parenchyma and collecting duct) and G4 (Conventional PN with only 0.5 ml peptide solution application) served as the control groups for the comparison of ABS and ABS nanohemostat-induced renal tissue effects. In the both control groups; although glomerulus are generally normal, fenestrates are spacious in some place of basal lamina on the side of the endothel and edema present. Pedicels are seen normal (Fig. 4). Though proximal tubules are seen normal in most of the field, intracellular edema is seen in some places (Fig. 5).

## Discussion

The efficacy of ABS was demonstrated in many studies. In a study which was conducted with 160 patients, it was shown that ABS is effective for gingival bleedings (Amer et al. 2014). In another study which was conducted by 25 patients who were undergone bronchoscopy procedure, ABS has successfully controlled the bleeding episodes of the cases (Uzun et al. 2014). There are three different clinical studies which confirms ABS as a successful hemostatic agent that was conducted with 25, 50 and 113 patients, respectively (Kazancıoğlu et al. 2013; Tek et al. 2014; Beyazit et al. 2011). ABS could also be used in gynecological cases. For example, in a study that was conducted on 40 cases with episiotomy, ABS again controlled the bleeding episodes (Eyi et al. 2013). In the literature there are some studies that was conducted on younger population also. For example, oral hemorrhagic episodes of 12 children were controlled by ABS in a study (Leblebisatan et al. 2012). Also, in a study with 47 children, the tonsillectomy procedures were successfully completed with ABS (Teker et al. 2009). Gastrointestinal and thyroidal tissue is also the sites of use for ABS. Hemorrhagic episodes in 30 cases who were performed gastric endoscopy and 61 cases who were undergone total thyroidectomy, were taken under control with ABS (Karaman et al. 2012; Guler et al. 2011). Otorhinolaryngology is another area that ABS is used. In two different studies which was conducted with 90 and 60 cases who were undergone adenoidectomy, the operations were performed without any complication with ABS (Iynen et al. 2011; Yasar and Ozkul 2011). The most astonishing reports regarding ABS are the emergent bypass procedures. There are two studies regarding this issue. In both studies 25 patients were included and ABS is shown to reduce bleedings significantly (Atalay et al. 2015; Akpinar et al. 2015). Apart from these clinical efficiency data, unfortunately in the literature there are limited data regarding safety in tissue level. Also ABS induced vital erythroid aggregation was never shown at ultrastructural level. In previously published clinical studies, the confirmation of safety issue in tissue level is a compelling problem since there are ethical obstacles about acquiring samples of patient’s tissues in clinical conditions of emergency and hemorrhage.

ABS nanohemostat is chimeric molecule that was synthesized by combining a self-assembling peptide amphiphile (PA) molecule with the traditional Ankaferd hemostat. The efficacy of ABS nanohemostat was shown previously in an experimental partial nephrectomy model (Huri et al. 2013). In our study, we have observed the erythroid aggregation in previously collected renal tissue. Moreover, we have observed no tissue damage by ABS nanohemostat including necrosis.

Although there is no ultrastructural analysis of the effect of ABS in the literature previously, the effect of ABS on blood vessels have already been elegantly demonstrated under the light microscopy by Saçak et al. (2014). In their study, the hemostatic effect of ABS on the vascular tissue were investigated. ABS effect of preventing the microvascular leakage on an anastomosis site and its long-term impact had been demonstrated. Although the mean bleeding time of the ABS group in that study was shorter, the pseudo-aneurysm formation on the vascular tissue was observed three weeks after the operation. Histological examination revealed the increased inflammatory cell infiltration, tunica media degeneration and contraction of the tunica intima in ABS group (Saçak et al. 2014).

We had four study groups in this research. SEM analyses of G1 (Left PN with hilar vascular control including intracorporeal suturing of the renal parenchyma and collecting duct) and G4 (Conventional PN with only 0.5 ml peptide solution application) disclosed comparable intracellular edema. Both of the G1 and G4 groups were served as the controls in order to test the effects of ABS and ABS nanohemostat at the renal tissue level. The investigation groups namely; G3 [Conventional PN with ABS (0.25 ml) + peptide (0.25 ml) gel (ABS Nanohemostat) mixture application with no suturing] and G4 (Conventional PN with only 0.5 ml peptide solution application) were the main research focus in this study. SEM analyses of the G3 and G4 study groups revealed the significant finding that erythroid aggregation are present inside the capillary bed of the renal tissue with the topical application of both ABS and ABS nanohemostat. The main hemostatic action of vital erythroid aggregation upon the exposure of ABS onto tissues has been elucidated via SEM analyses at the renal tissue level in this present study. Since the main supposed function of peptide amphiphile (PA) molecule is to carry active ABS onto the tissue compartments, its addition did not effected the hemostatic action of ABS and served just as a ‘drug carrier’. Erythroid aggregation is provided by erythroid receptors and proteins (spectrin, ankyrin, actin) which is fixed by functional proteomics of ABS (Demiralp et al. 2010). Erythrocyte aggregation tightens on fibrinogen gamma and resulting erythroid network is provided to interact with the vascular endothelium (Demiralp et al. 2012). Because gamma fibrinogen has antithrombin I effect, a coagulation-axis thrombotic conditions and endothelial damage does not evolved in this process. At the level of cellular and intracellular organelles ABS damage does not develop. The repair of hurtful process secondary to trauma is carried out by physiological hemostasis. Edema following the use of topical hemostatic agents is thought to be associated with endothelial activation. Urotensin II which is inside ABS can be held responsible in this situation (Demiralp et al. 2012). Tubular edema development following the use of topical hemostatic agents is thought to be related to the renal tubular apoptosis which is previously shown by Huri et al. (2009).

Our study is important for several reasons. Firstly, in our study we used ABS nanohemostat which was recently developed. There are very few data regarding ABS nanohemostat. Our study adds valuable information to the literature regarding ABS nanohemostat. Secondly, this is the first study that reveals the ultrastructural analysis of ABS at tissue level. Previously, ultrastructural analysis of ABS was only performed in blood samples in the literature. There are no electron microscopy observations regarding ABS at tissue level. Thirdly, first time in the literature we detected ABS nanohemostat induced vital erythroid aggregation at tissue level by SEM. Lastly and the most important finding of our study is that we detected ABS nanohemostat causes no harm to tissues including necrosis and any other effects. This is the first ultrastructural analysis of ABS nanohemostat regarding the safety issue at tissue level. By these findings in our study we confirm that ABS nanohemostat is a safe hemostatic agent and could be used especially in the renal tissue without any doubts. However there are still many efforts to spend in order to increase our knowledge about ABS nanohemostat. Phase 1, 2 and 3 studies should be performed regarding ABS nanohemostat since this agent should be considered as a new agent apart from traditional ABS. Moreover, we have revealed the efficient and safety of ABS nanohemostat in renal tissue; however these effects should be confirmed in other tissues by other studies. Our findings may be the starting point for upcoming future studies which would add valuable information about ABS nanohemostat.
